# Accuracy of shear wave elastography for the diagnosis of prostate cancer: A meta-analysis

**DOI:** 10.1038/s41598-017-02187-0

**Published:** 2017-05-16

**Authors:** Liang Sang, Xue-mei Wang, Dong-yang Xu, Yun-fei Cai

**Affiliations:** 1 Ultrasound Department of the First Hospital of China Medical University, Shenyang, 110001 China; 2 Dermatology Department of the First Hospital of China Medical University, Shenyang, 110001 China

## Abstract

Many studies have established the high diagnostic accuracy of shear wave elastography (SWE) for the detection of prostate cancer (PCa); however, its utility remains a subject of debate. This meta-analysis sought to appraise the overall accuracy of SWE for the detection of PCa. A literature search of the PubMed, Embase, Cochrane Library, Web of Science and CNKI (China National Knowledge Infrastructure) databases was conducted. In all of the included studies, the diagnostic accuracy of SWE was compared with that of histopathology, which was used as a standard. Data were pooled, and the sensitivity, specificity, area under the curve (AUC), positive likelihood ratio (PLR), negative likelihood ratio (NLR), and diagnostic odds ratio (DOR) were calculated to estimate the accuracy of SWE. The pooled sensitivity and specificity for the diagnosis of PCa by SWE were 0.844 (95% confidence interval: 0.696–0.927) and 0.860 (0.792–0.908), respectively. The AUC was 0.91 (0.89–0.94), the PLR was 6.017 (3.674–9.853), and the NLR was 0.182 (0.085–0.389). The DOR was 33.069 (10.222–106.982). Thus, SWE exhibited high accuracy for the detection of PCa using histopathology as a diagnostic standard. Moreover, SWE may reduce the number of core biopsies needed.

## Introduction

Prostate cancer (PCa) is a public health problem worldwide. PCa is the most common malignant tumor in adult males, and the incidence rate is increasing^1^. Moreover, PCa is the second leading cause of cancer death in men. Recently, diagnoses of PCa have relied on levels of prostate-specific antigen (PSA) and digital rectal examination (DRE), although pathologic histology remains the gold standard. However, these approaches potentially lead to under-diagnosis of PCa, whereas biopsy is an invasive method associated with patient discomfort and, in some cases, serious complications.

Although traditional grayscale transrectal ultrasonography (TRUS) is routinely used in diagnosis and to guide biopsy, it is not sufficiently sensitive or specific for biopsy procedures^2^. Biopsy protocols should be optimized to accurately detect PCa while also reducing the number of prostate biopsy specimens and biopsy-related patient morbidity^3^. The prostate gland is one of the earliest organs for which elastography was proposed and applied^4^. PCa is stiffer than normal tissue because of its increased cellularity, which is sometimes found during DRE^5–7^. Transrectal elastosonography (TRES) has already been established to be feasible in guiding biopsies and for improving the detection of prostate lesions^8–11^.

Shear wave elastography (SWE) is a novel real-time imaging technique that represents a substantial advance in ultrasound elastography. When SWE is performed, the transducer automatically generates acoustic radiation force using a special “supersonic” speed that moves multiple focus points following the Mach cone principle. Tissue is then mechanically excited by the Mach cone impulse to generate small, localized tissue displacements (1–10 mm). These displacements have been tracked using a system to calculate the shear wave propagation speed and the quantitative tissue stiffness (i.e., Young’s modulus, kPa)^12, 13^, which is defined as E = σ/ε, where σ is the applied stress and ε is the strain (the ratio of the resultant deformation of tissue over the original reference length of the medium)^14^. Previous studies have established that the Young’s modulus of PCa was significantly greater than that of benign prostatic tissue; the sensitivity ranged from 43% to 96.2%, and the specificity ranged from 69.1% to 96.2%^14–20^. However, there have been large differences among the results of different studies, and the cut-off value for adequate distinction between PCa and benign tissue remains undetermined. Additionally, another study found that SWE was a poor predictor of malignancy and that the cut-off value had little practical meaning^21^. Therefore, the present study aimed to perform a meta-analysis to appraise the overall accuracy of SWE for the diagnosis of PCa.

## Results

### Characteristics of the included studies

**Figure 1:**
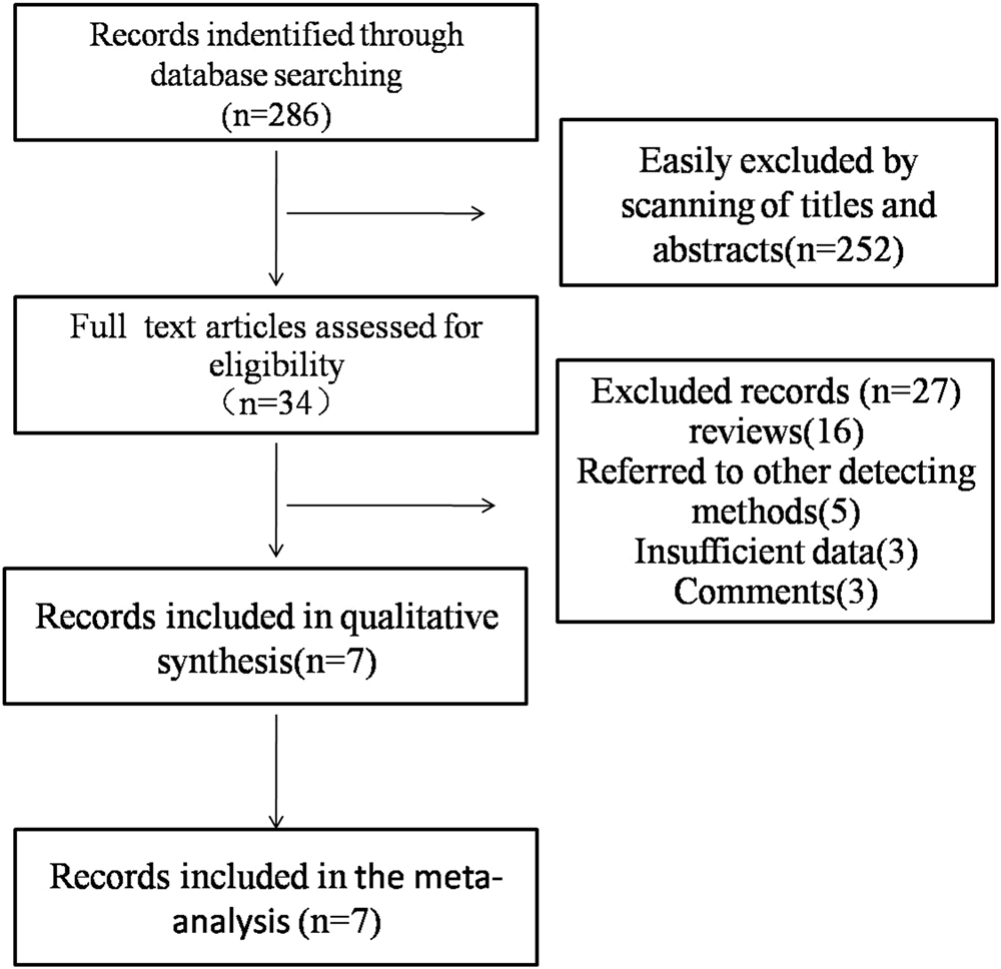
Literature search and selection scheme.

An electronic search identified 286 records. After screening titles and abstracts, we identified 34 studies for full text review. Among these articles, we classified 16 as review articles, 3 as comments, and 5 as other detection methods, whereas 3 had insufficient data for calculation. Ultimately, 7 correlative studies were identified as eligible studies; these were published from 2012 to 2016 (Fig. 1). Among those studies, two were analyzed twice, one according to PSA groupings of 4–20 μg/L and over 20 μg/L and one according to the ultrasonography section (axial vs. sagittal). Ultimately, data from 9 groups were included in this meta-analysis. Additionally, only one study referred to the transition zone, which is rare for PCa, and partial data were not included in the statistical analysis. Among these studies, 2 evaluated the diagnostic accuracy of SWE compared with the histopathology of radical prostatectomy (RP) specimens as a reference standard, whereas 5 compared SWE with the histopathology of TRUS biopsy specimens. The patient clinical features and essential data are summarized in Table 1.

**Table 1:** Characteristics of the included studies.

	First author	Year	Country	Age (avg)	PSA (μg/L)	Number of patients	Number of samples	Ultrasound system	Cut-off value (kPa)	TP	FN	FP	TN
1	Zhang Mo	2015	China	70.21	14.52	489	NA	Supersonic Imagine Aixplorer	28.5	196	25	37	231
2.1	Sarfraz Ahmad	2013	UK	69	4–20	39	485	SuperSonic Imagine, Aix-en-Provence	NA	286	29	20	150
2.2	Sarfraz Ahmad	2013	UK	69	>20	11	141	SuperSonic Imagine, Aix-en-Provence	NA	102	7	2	30
3.1	Olivier Rouvière	2016	France	63	6.5	NA	251	SuperSonic Imagine, Aix-en-Provence	45	45	40	18	148
3.2	Olivier Rouvière	2016	France	63	6.5	NA	227	SuperSonic Imagine, Aix-en-Provence	52	44	28	46	109
4	Richard G. Barr	2012	America	64.2	5.05	53	318	SuperSonic Imagine, Aix-en-Provence	37	25	1	11	281
5	Katharina Boehm	2014	Germany	NA	8.7	60	322	Aixplorer System	50	114	27	56	125
6	Sungmin Woo	2014	Korea	66	12.8	87	1058	SuperSonic Imagine, Aix-en-Provence	43.9	34	45	188	791
7	Jean-Michel Correas	2015	France	65.1	7.4	184	1040	SuperSonic Imagine	35	124	5	137	774

Age (Avg.) = Average age of patients; TP = True positive; FN = False negative; FP = False positive; TN = True negative. Data from one study were divided into two groups according to the PSA level: 2.1 (4–20 μg/L) and 2.2 (over 20 μg/L). Data from the other studies were divided into two groups according to the ultrasonography section: 3.1 (axial section) and 3.2 (sagittal section).

### Methodological quality assessment of the included studies

Quality evaluation results for the individual studies are shown in Table 2. The overall risk of bias was low because the index test and reference test characterization were adequate in most studies, and only one equivocal result was reported. In two studies^15, 17^, it was unclear whether the pathologist was blinded to the SWE results. One study^14^ used a previously determined cut-off value, which was based on clinical experience and reported in the literature as the SWE reference standard. Another study^19^ found that SWE was limited as a tool to reliably differentiate benign from malignant prostate tissues.

**Table 2:** Quality assessment of the included studies.

First Author	Risk of Bias				Applicability Concerns		
	Patient Selection	Index Test	Reference Standard	Flow and Timing	Patient Selection	Index Test	Reference Standard
Zhang Mo	low	low	unclear	low	low	low	low
Sarfraz Ahmad	low	high	low	low	low	low	low
Olivier Rouvière	low	low	low	low	low	low	low
Richard G. Barr	low	low	unclear	low	low	low	low
Katharina Boehm	low	low	low	low	low	low	low
Sungmin Woo	low	low	low	low	low	unclear	low
Jean-Michel Correas	low	low	low	low	low	low	low

The table summarizes the risk of bias and applicability concerns.

### Diagnostic accuracy

**Figure 2:**
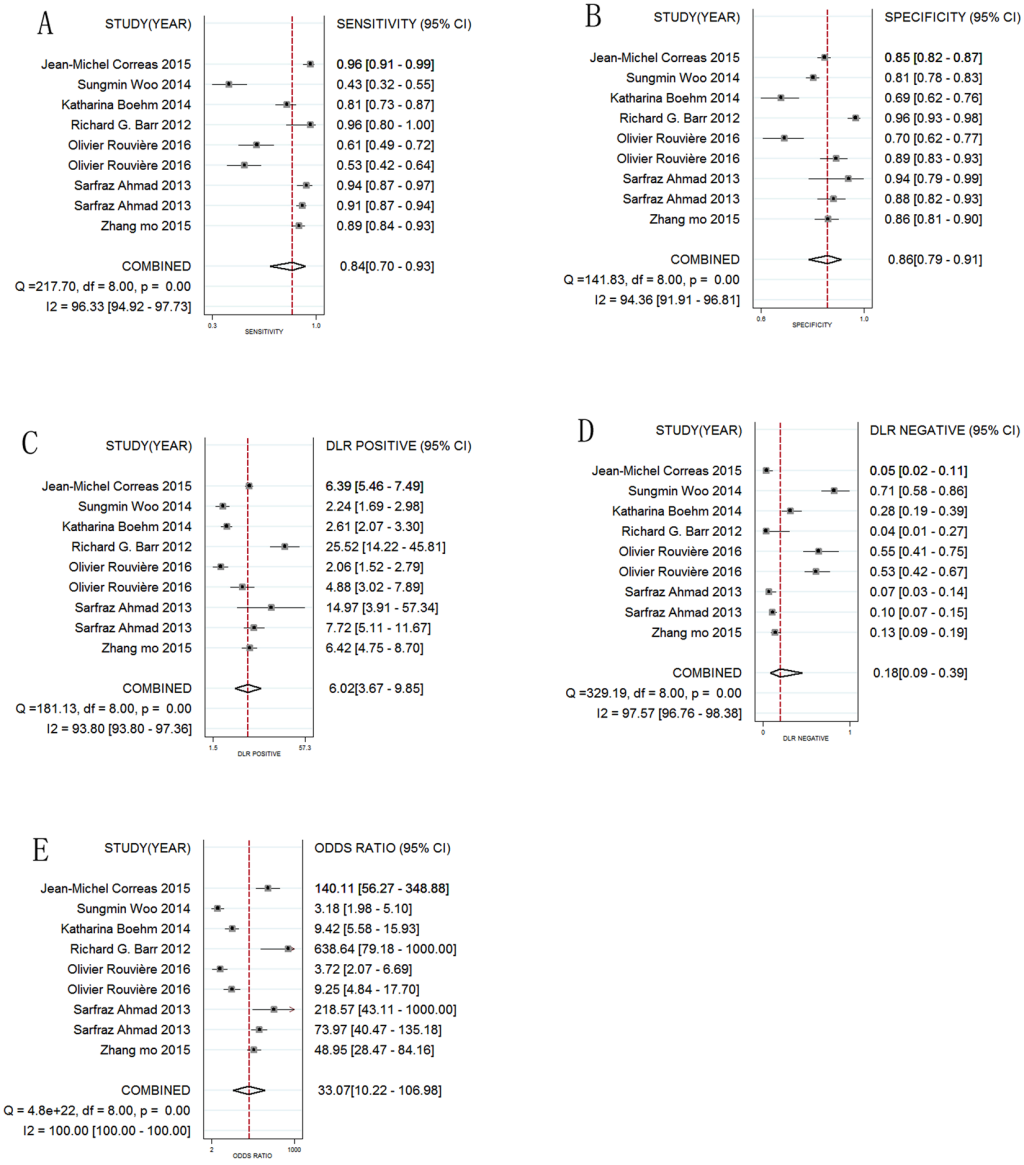
Forest plots of the diagnostic accuracy of SWE in PCa. A = Sensitivity; B = Specificity; C = Positive likelihood ratio; D = Negative likelihood ratio; E = Diagnostic odds ratio; CI = Confidence interval; LR = Likelihood ratio.

**Figure 3:**
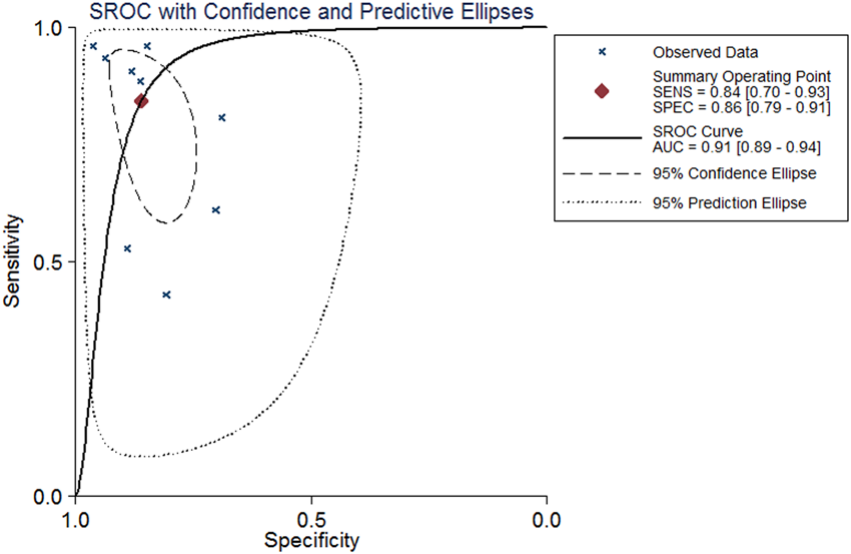
Summary receiver operating characteristic (SROC) curve for SWE in the diagnosis of PCa for all studies. AUC = Area under the curve.

**Figure 4:**
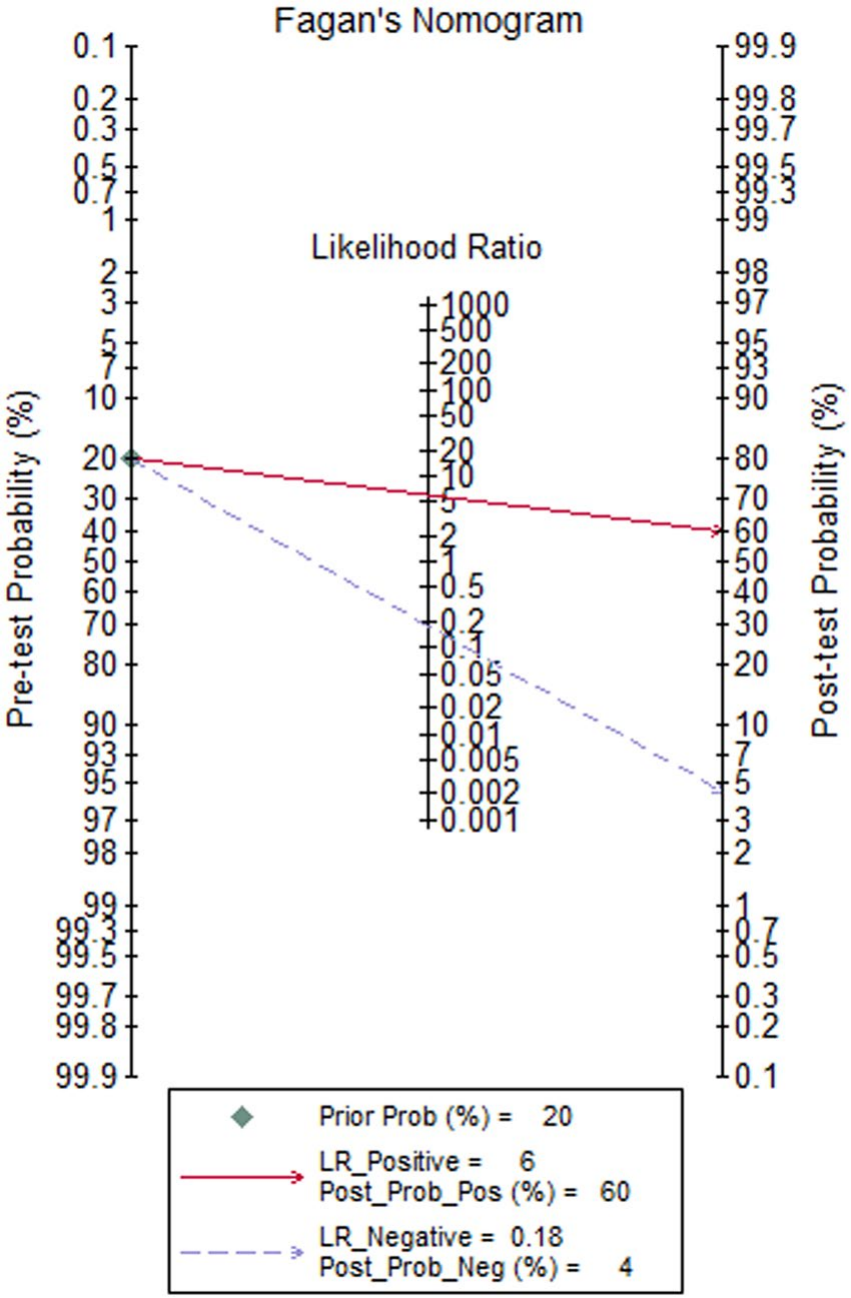
Result of Bayesian analyses showing the pre- and post-test likelihoods for PCa detection. The pre-test probability is the probability of a PCa episode being detected without taking SWE into account. The post-test probability takes into account the results of SWE. LR = Likelihood ratio.

Statistical analysis revealed no heterogeneity arising from a threshold effect, and the Spearman correlation coefficient of sensitivity and 1-specificity was −0.533 (p = 0.139). Ultimately, the diagnostic accuracy of SWE for the diagnosis of PCa was computed based on a pooled sensitivity of 0.844 (95% confidence interval (CI): 0.696–0.927), pooled specificity of 0.860 (95% CI: 0.792–0.908), pooled positive likelihood ratio (PLR) of 6.017 (95% CI: 3.674–9.853), pooled negative likelihood ratio (NLR) of 0.182 (95% CI: 0.085–0.389), and pooled diagnostic odds ratio (DOR) of 33.069 (95% CI: 10.222–106.982). Forest plots of all indices are shown in Fig. 2. An overall high degree of accuracy was revealed by the summary receiver operating characteristic (SROC) curve with an area under the curve (AUC) of 0.91 (95% CI: 0.89–0.94) (Fig. 3). A Fagan nomogram was constructed to illustrate the pre- and post-test probability of SWE to predict PCa based on all 7 studies (Fig. 4). Without taking into account the results of SWE, a PCa episode had a ‘pre-test’ probability of 20% to be detected. With a SWE-positive result for the detection of PCa, there was a 60% ‘post-test’ probability of a subsequent PCa episode. With a negative SWE, the ‘post-test’ probability of PCa dropped to 4%.

### Evaluation of publication bias

**Figure 5:**
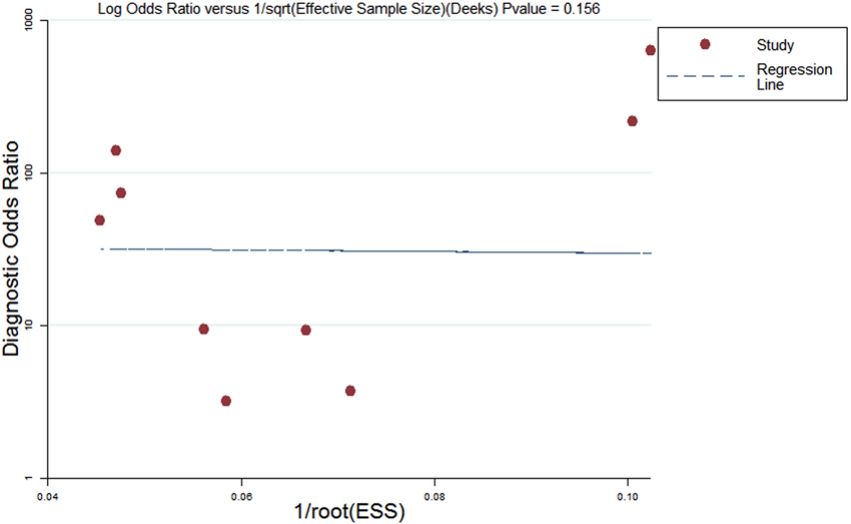
Funnel plot for the evaluation of potential publication bias. Each solid circle represents a study in the meta-analysis. The line is the regression line.

A Deeks’ funnel plot was generated to explore the potential for publication bias. Based on the symmetric shape of the funnel plot of the pooled DOR (Fig. 5) and the Deeks’ test non-significant value (p = 0.156), we detected no potential publication bias in this meta-analysis.

## Discussion

Currently, several methods are used to detect PCa. According to current guidelines^22^, diagnosis should include PSA level measurement, DRE and TRUS. However, none of these measurements can provide an optimal diagnosis for PCa because of limitations of each approach. PSA has led to many cases of misdiagnosis due to its high sensitivity but low specificity^23^, resulting in many patients with benign lesions undergoing unnecessary biopsy^23, 24^. DRE has been used as a screening tool for PCa; however, DRE is examiner-dependent method and is limited to the posterior part of the prostate. TRUS is a safe procedure that can provide effective evidence for the detection of PCa. Unfortunately, TRUS is a non-quantitative method that is associated with subjective measurements and largely depends upon the ability of the physician performing the examination; it has a reported sensitivity of 17–57% and specificity of 40–63%^25^. Therefore, developing an ideal imaging and detection method for PCa that offers high overall sensitivity and specificity is essential.

An increased cell density of a neoplastic mass leads to changes in tissue elasticity such that the stiffness of normal tissue is significantly different from that of tumor tissue^26, 27^. Elastography is an imaging technique used for the detection of cancer tissue based on stiffness differences among various tissues^28^, and it has been shown to be a useful diagnostic method for many organs, such as the thyroid, breast and prostate^29–31^. Most studies have reported a remarkable amelioration in PCa identification using elastography^32, 33^. The sensitivity of elastography for PCa diagnosis can reach or exceed 90%, which is obviously greater than that of PSA, DRE or TRUS^32, 34, 35^. However, traditional elastography also has many limitations, mostly due to the lack of uniform repeatability resulting from manual compression and operator dependency, which can introduce extensive variability^36–38^.

SWE is a technique that uses a sonographic pulse to produce a shear wave in the tissue^39, 40^. Tissue stiffness is expressed as the Young’s modulus or simply as the ratio of stress generated by tissue deformation^41^. A previous study showed no significant difference in intra-observer reproducibility among the measurements stratified by prostate gland volume, patient age, or levels of serum PSA^42^. Compared with quasistatic compression elastography, SWE is much closer to a standard TRUS clinical examination because it does not require any additional compression.

Recently, SWE has been shown to be a useful technique for prostate examination^9, 14–19, 21, 42^. Barr *et al*.^17^ reported that SWE showed a high sensitivity of 96.2%, specificity of 96.2%, positive predictive value (PPV) of 69.4%, and negative predictive value (NPV) of 99.6% for the detection of PCa when 37 kPa was used as a cut-off value between benign and malignant lesions. Ahmad *et al*.^14^ also showed that the sensitivity and specificity of SWE for PCa detection could each reach 90%. However, Woo *et al*.^19^ reported low sensitivity and variable specificity for the diagnostic value of SWE in the detection of PCa, even though the SWE parameters were significantly different between PCa and benign prostate tissues. Additionally, Porsch *et al*.^21^ showed that SWE was a poor predictor of malignancy for prostate lesions. Considering these inconsistent results, we believed it necessary to assess the diagnostic value of SWE for the detection of PCa. To the best of our knowledge, this represents the first meta-analysis to evaluate the diagnostic value of SWE for the detection of PCa.

Literature screening was carried out following a strict protocol, and the search ultimately identified 7 relevant studies. Deeks’ funnel plots showed no significant publication bias, and according to the QUADAS-2 questionnaire, the 7 studies were of high quality. Our results showed that SWE had a pooled sensitivity of 84.4% and specificity of 86.0% for the detection of PCa; these values are both higher than those obtained for traditional TRUS^32^ and real-time elastography for the diagnosis of patients with suspected PCa^37^. The AUC (0.91) and DOR (33.069) further indicated perfect overall accuracy. Additionally, the PLR value was 6.017 (95% CI: 3.674–9.853), which was clinically meaningful for our measures of diagnostic accuracy.

Currently, the success rate of systematic prostate biopsy varies from 25% to 30%, whereas its false-negative rate ranges from 17% to 21% in patients with a negative initial series of biopsies^43, 44^. Real-time quantitative SWE imaging has the potential to change the clinical practice of PCa identification and screening by improving the localization of abnormal foci and allowing limited targeted biopsies of suspicious areas, thereby reducing both complications and costs associated with the current standard of care^14^. Although there was no cut-off-value-related heterogeneity in this meta-analysis, it would be of interest to determine whether the measured stiffness or a specific cut-off value predicts up- or down-grading of these regions. This topic could be the subject of future investigations.

A comprehensive literature search and careful data extraction were performed to avoid bias. Nevertheless, limitations exist in our study. First, we did not carry out subgroup analysis of patients with different measurement locations; previous studies have revealed that the location of tumor foci within the prostate gland can influence the detection rate using TRES^5, 16, 45, 46^. Although SWE provides much-needed solutions to the ongoing challenge of accurately locating areas of interest in the prostate, it also has the inherent advantage of independence from operator experience and expertise. Second, most studies considered in this meta-analysis used TRUS-guided biopsy data as a reference standard for PCa detection, whereas two studies used histopathology analyses of RP specimens. Although TRUS-guided biopsy is the recommended diagnostic method for most patients suspected of having PCa^47^, this method performs poorly in locating PCa compared with histopathology of the RP specimen^48^, and SWE estimates also lack strong correlations with PCa location. Third, we failed to acquire unpublished data, and language limitations might have affected the reliability of our results. Fourth, this meta-analysis did not evaluate the correlation between the stiffness value of a lesion and the Gleason score because of a lack of valid data for extraction despite the fact that the Gleason score is one of the most frequently used histologic grading systems for PCa^49^.

Based on the findings of this meta-analysis and previous studies, we consider SWE to be a novel and non-invasive imaging technique that is superior to conventional TRUS for the assessment of tissue stiffness to provide information for the detection of PCa and biopsy guidance. The application of SWE might lead to a decrease in the number of biopsy cores. Although SWE does not require any additional compression compared with quasistatic compression elastography and no significant difference in intra-observer reproducibility among the measurements^42^, practitioners should be trained in its application, and reference standards should be agreed upon for the location of prostate cancer lesions and histopathology. The Gleason score is one of the most frequently used histologic grading systems for PCa, and the prognosis of PCa is closely related to the Gleason score^49^; thus, multicenter studies with a larger number of cases should be conducted to reveal the correlation between the Gleason score and the tissue stiffness of PCa. In addition, a previous study^50^ showed that multiparametric MRI (mpMRI) provided the best anatomical and functional imaging of the prostate compared with that of other imaging methods, and a systematic review^51^ suggested that mpMRI could be used to trigger a targeted repeat biopsy for prostate cancer diagnosis. Future research should be performed to evaluate the correlations between SWE and mpMRI with histopathology as the gold standard.

In conclusion, this meta-analysis shows that SWE has high sensitivity and specificity for the detection of PCa and is useful for differentiating between malignant and benign prostate lesions. Thus, we believe that SWE could improve the guiding capability and reduce the unnecessary core biopsies required for diagnosis. Further studies with a multicenter design will be needed to assess the role of SWE in the detection of PCa.

## Methods

### Search strategy

An independent search of the English and Chinese medical literature using the PubMed (Medicine) database and cross-citation with other databases (i.e., Embase, Cochrane Library databases, Web of Science and CNKI) was performed to identify all studies involving diagnostic tests that estimated the value of SWE for the diagnosis of PCa. Searches were conducted using the following key words: elastography, sonoelastography, and elastosonography combined with prostate. Repeated articles were manually excluded. Unpublished relevant data were also considered, but no studies with such data were found that were appropriate for inclusion. This study was performed by two independent authors. The search was updated until October 23, 2016.

### Eligibility and exclusion criteria

All articles were evaluated independently by two authors. A study was included if it met the following criteria: (1) a cross-sectional study that evaluated the ability of SWE to detect PCa; (2) use of histopathology as a diagnostic standard; and (3) reported data (sensitivity and specificity) necessary to calculate the true-positive, false-negative, false-positive and true-negative rates of SWE in the diagnosis of PCa. All of the included studies should have obtained informed consent from study participants and received protocol approval by an ethics committee or institutional review board. Review articles, conference reports, letters, editorial comments, opinions, prefaces, low-quality studies and articles not published in English or Chinese were excluded. All disagreements were resolved by consensus.

### Data extraction

All relevant data from the 7 included studies, including first author; year that the study was performed; age of subjects; PSA level; number of patients; number of samples; ultrasound system; cut-off value; and number of true positives, false negatives, false positives and true negatives, were extracted in a unified form. Any divergence from this procedure was resolved by discussion.

### Assessments of methodological quality

Methodological quality was evaluated using the revised Quality Assessment of Diagnostic Accuracy Studies (QUADAS-2)^52^ included in a systematic reviews tool. QUADAS-2 classifies risks for bias into four key domains that encompass patient selection, index test, reference standard, flow and timing. Each domain was assessed in terms of the risk of bias, and patient selection, index test, and reference standard were also assessed for applicability. Two authors independently conducted the quality assessment, and any disagreements were resolved by discussion or appeal to a third author.

### Statistical analysis

The statistical software package STATA, version 11.0 (Stata Corporation, College Station, TX, USA), and Meta-Disc, version 1.4 for Windows (XI Cochrane Colloquium, Barcelona, Spain), were used in this study. To research possible heterogeneity resulting from the threshold effect, we calculated Spearman correlation coefficients between sensitivity and 1-specificity. The pooled sensitivity, specificity, AUC, PLR, NLR, DOR, and other related indexes were calculated using STATA. Fagan’s nomogram was used to visualize the detection of SWE for PCa using likelihood ratios to calculate a post-test probability based on Bayesian theorems. We performed Deeks’ funnel plot analysis to check for potential publication bias in our study, with a p*-*value < 0.1 suggesting statistical significance^53^.

## References

[1] Jemal A (2011). Global cancer statistics. CA: a cancer journal for clinicians.

[2] Bjurlin MA (2013). Optimization of initial prostate biopsy in clinical practice: sampling, labeling and specimen processing. The Journal of urology.

[3] Rodriguez LV, Terris MK (2000). Risks and complications of transrectal ultrasound. Current opinion in urology.

[4] Parker KJ, Huang SR, Musulin RA, Lerner RM (1990). Tissue response to mechanical vibrations for “sonoelasticity imaging”. Ultrasound in medicine & biology.

[5] Salomon G (2008). Evaluation of prostate cancer detection with ultrasound real-time elastography: a comparison with step section pathological analysis after radical prostatectomy. European urology.

[6] Konig K (2005). Initial experiences with real-time elastography guided biopsies of the prostate. The Journal of urology.

[7] Ophir J (1999). Elastography: ultrasonic estimation and imaging of the elastic properties of tissues. Proceedings of the Institution of Mechanical Engineers. Part H, Journal of engineering in medicine.

[8] Sumura M (2007). Initial evaluation of prostate cancer with real-time elastography based on step-section pathologic analysis after radical prostatectomy: a preliminary study. International journal of urology: official journal of the Japanese Urological Association.

[9] Brock M (2012). The impact of real-time elastography guiding a systematic prostate biopsy to improve cancer detection rate: a prospective study of 353 patients. The Journal of urology.

[10] van Hove A (2014). Comparison of image-guided targeted biopsies versus systematic randomized biopsies in the detection of prostate cancer: a systematic literature review of well-designed studies. World journal of urology.

[11] Postema A, Mischi M, de la Rosette J, Wijkstra H (2015). Multiparametric ultrasound in the detection of prostate cancer: a systematic review. World journal of urology.

[12] Bercoff J, Tanter M, Fink M (2004). Supersonic shear imaging: a new technique for soft tissue elasticity mapping. IEEE transactions on ultrasonics, ferroelectrics, and frequency control.

[13] Franchi-Abella S, Elie C, Correas JM (2013). Ultrasound elastography: advantages, limitations and artefacts of the different techniques from a study on a phantom. Diagnostic and interventional imaging.

[14] Ahmad S, Cao R, Varghese T, Bidaut L, Nabi G (2013). Transrectal quantitative shear wave elastography in the detection and characterisation of prostate cancer. Surgical endoscopy.

[15] Mo Z (2015). Transrectal shear wave elastography combined with transition zone biopsy for detecting prostate cancer. Zhonghua Nan Ke Xue Za Zhi.

[16] Rouviere O (2016). Stiffness of benign and malignant prostate tissue measured by shear-wave elastography: a preliminary study. European radiology.

[17] Barr RG, Memo R, Schaub CR (2012). Shear wave ultrasound elastography of the prostate: initial results. Ultrasound quarterly.

[18] Boehm K (2015). Shear wave elastography for localization of prostate cancer lesions and assessment of elasticity thresholds: implications for targeted biopsies and active surveillance protocols. The Journal of urology.

[19] Woo S, Kim SY, Cho JY, Kim SH (2014). Shear wave elastography for detection of prostate cancer: a preliminary study. Korean journal of radiology.

[20] Correas JM (2015). Prostate cancer: diagnostic performance of real-time shear-wave elastography. Radiology.

[21] Porsch M (2015). New aspects in shear-wave elastography of prostate cancer. Journal of ultrasonography.

[22] Heidenreich A (2014). EAU guidelines on prostate cancer. part 1: screening, diagnosis, and local treatment with curative intent-update 2013. European urology.

[23] Borley N, Feneley MR (2009). Prostate cancer: diagnosis and staging. Asian journal of andrology.

[24] Aigner F (2011). Comparison of real-time sonoelastography with T2-weighted endorectal magnetic resonance imaging for prostate cancer detection. Journal of ultrasound in medicine: official journal of the American Institute of Ultrasound in Medicine.

[25] Seitz, M. *et al*. [Imaging procedures to diagnose prostate cancer]. *Der Urologe. Ausg. A* **46**, W1435–1446; quiz W1447–1438, doi:10.1007/s00120-007-1455-x (2007). 10.1007/s00120-007-1455-x 17665166

[26] Phipps S, Yang TH, Habib FK, Reuben RL, McNeill SA (2005). Measurement of tissue mechanical characteristics to distinguish between benign and malignant prostatic disease. Urology.

[27] Krouskop TA, Wheeler TM, Kallel F, Garra BS, Hall T (1998). Elastic moduli of breast and prostate tissues under compression. Ultrasonic imaging.

[28] Miyagawa T (2009). Real-time elastography for the diagnosis of prostate cancer: evaluation of elastographic moving images. Japanese journal of clinical oncology.

[29] Lyshchik A (2005). Thyroid gland tumor diagnosis at US elastography. Radiology.

[30] Itoh A (2006). Breast disease: clinical application of US elastography for diagnosis. Radiology.

[31] Rhymer, J. C. Elastography in the detection of prostatic cancer. *Clinical radiology* **58**, 337; author reply 337 (2003). 10.1016/s0009-9260(03)00065-5 12662961

[32] Brock M (2011). Comparison of real-time elastography with grey-scale ultrasonography for detection of organ-confined prostate cancer and extra capsular extension: a prospective analysis using whole mount sections after radical prostatectomy. BJU international.

[33] Dudea SM (2011). Value of ultrasound elastography in the diagnosis and management of prostate carcinoma. Medical ultrasonography.

[34] Miyanaga N (2006). Tissue elasticity imaging for diagnosis of prostate cancer: a preliminary report. International journal of urology: official journal of the Japanese Urological Association.

[35] Souchon R (2005). Monitoring the formation of thermal lesions with heat-induced echo-strain imaging: a feasibility study. Ultrasound in medicine & biology.

[36] Correas JM (2013). Ultrasound elastography of the prostate: state of the art. Diagnostic and interventional imaging.

[37] Zhang B (2014). Real-time elastography in the diagnosis of patients suspected of having prostate cancer: a meta-analysis. Ultrasound in medicine & biology.

[38] Good DW (2014). Elasticity as a biomarker for prostate cancer: a systematic review. BJU international.

[39] Barr RG (2012). Sonographic breast elastography: a primer. Journal of ultrasound in medicine: official journal of the American Institute of Ultrasound in Medicine.

[40] Correas JM (2013). Update on ultrasound elastography: miscellanea. Prostate, testicle, musculo-skeletal. European journal of radiology.

[41] Ophir J (2000). Elastographic imaging. Ultrasound in medicine & biology.

[42] Woo S, Kim SY, Lee MS, Cho JY, Kim SH (2015). Shear wave elastography assessment in the prostate: an intraobserver reproducibility study. Clinical imaging.

[43] Singh H (2004). Predictors of prostate cancer after initial negative systematic 12 core biopsy. The Journal of urology.

[44] Mian BM (2002). Predictors of cancer in repeat extended multisite prostate biopsy in men with previous negative extended multisite biopsy. Urology.

[45] Pallwein L (2008). Prostate cancer diagnosis: value of real-time elastography. Abdominal imaging.

[46] Tsutsumi M (2007). The impact of real-time tissue elasticity imaging (elastography) on the detection of prostate cancer: clinicopathological analysis. International journal of clinical oncology.

[47] Smith RA, Cokkinides V, Eyre HJ (2007). Cancer screening in the United States, 2007: a review of current guidelines, practices, and prospects. CA: a cancer journal for clinicians.

[48] Muntener M (2008). Prognostic significance of Gleason score discrepancies between needle biopsy and radical prostatectomy. European urology.

[49] Barr RG (2017). WFUMB Guidelines and Recommendations on the Clinical Use of Ultrasound Elastography: Part 5. Prostate. Ultrasound in medicine & biology.

[50] Pokorny MR (2014). Prospective study of diagnostic accuracy comparing prostate cancer detection by transrectal ultrasound-guided biopsy versus magnetic resonance (MR) imaging with subsequent MR-guided biopsy in men without previous prostate biopsies. European urology.

[51] Haider MA, Yao X, Loblaw A, Finelli A (2016). Multiparametric Magnetic Resonance Imaging in the Diagnosis of Prostate Cancer: A Systematic Review. Clinical oncology.

[52] Whiting PF (2011). QUADAS-2: a revised tool for the quality assessment of diagnostic accuracy studies. Annals of internal medicine.

[53] Deeks JJ, Macaskill P, Irwig L (2005). The performance of tests of publication bias and other sample size effects in systematic reviews of diagnostic test accuracy was assessed. Journal of clinical epidemiology.

